# Acceptability of innovative culture-based antibiotic prophylaxis strategies: a multi-method study on experiences regarding transrectal prostate biopsy

**DOI:** 10.1093/jacamr/dlab161

**Published:** 2021-11-17

**Authors:** Sofie C M Tops, Anita M P Huis, Willeke Trompers, Anke J M Oerlemans, J P Michiel Sedelaar, Eva Kolwijck, Heiman F L Wertheim, Marlies E J L Hulscher

**Affiliations:** 1 Department of Medical Microbiology, Center for Infectious Diseases, Radboud University Medical Center, Nijmegen, The Netherlands; 2 Scientific Center for Quality of Healthcare (IQ healthcare), Radboud Institute for Health Sciences, Radboud University Medical Center, Nijmegen, The Netherlands; 3 Department of Urology, Radboud University Medical Center, Nijmegen, The Netherlands; 4 Department of Medical Microbiology, Jeroen Bosch Hospital, ’s-Hertogenbosch, The Netherlands

## Abstract

**Background:**

The acceptability of innovative medical strategies among healthcare providers and patients affects their uptake in daily clinical practice.

**Objectives:**

To explore experiences of healthcare providers and patients with culture-based antibiotic prophylaxis in transrectal prostate biopsy with three swab-screening scenarios: self-sampling at home, self-sampling in the hospital and sampling by a healthcare provider.

**Methods:**

We performed focus group interviews with urologists and medical microbiologists from 11 hospitals and six connected clinical microbiological laboratories. We used Flottorp’s comprehensive checklist for identifying determinants of practice to guide data collection and analysis. The experiences of 10 laboratory technicians from five laboratories and 452 patients from nine hospitals were assessed using a questionnaire.

**Results:**

Overall, culture-based prophylaxis strategies were experienced as feasible in daily clinical practice. None of the three swab-screening scenarios performed better. For urologists (*n = *5), implementation depended on the effectiveness of the strategy. In addition, it was important to them that the speed of existing oncology care pathways is preserved. Medical microbiologists (*n = *5) and laboratory technicians (*n = *8) expected the strategy to be fairly easy to implement. Patients (*n = *430; response rate 95.1%) were generally satisfied with the screening scenario presented to them. To meet the various patients’ needs and preferences, multiple scenarios within a hospital are probably needed.

**Conclusions:**

This multi-method study has increased our understanding of the acceptability of culture-based prophylaxis strategies in prostate biopsy, which can help healthcare providers to offer high-quality patient-centred care. The strategy seems relatively straightforward to implement as overall acceptance appears to be high.

## Introduction

Antimicrobial resistance (AMR) to antibiotics is a serious global public health problem.^1^ Many medical procedures depend on the availability of effective antibiotic prophylaxis to reduce infectious complications after these procedures.^1^ To curb AMR, culture-based prophylaxis might be applied to reduce the use of inappropriate (broad-spectrum) antibiotic prophylaxis.^2^^,^^3^ Also, the presumed reduction of infectious complications associated with this strategy will reduce the use of therapeutic antibiotics, which will again contribute to reducing the AMR threat.

One of the procedures in which culture-based prophylaxis could be a promising strategy is transrectal prostate biopsy (PB). Transrectal PB, which is used to diagnose and stage prostate cancer, is one of the most frequently performed urological procedures.^4^ A worrying increase in infectious complications after transrectal PB from <1% to 6% now has been reported in recent years.^5–7^ This rise in post-biopsy infections has been linked to the growing resistance of Gram-negative bacteria, the main pathogens involved, to fluoroquinolone (FQ) antibiotics.^8^^,^^9^

Two meta-analyses have provided evidence for the use of culture-based prophylaxis in reducing infectious complications after transrectal PB.^3^^,^^10^^,^^11^ One meta-analysis from 15 studies representing 12 320 participants, showed infectious complications in 3.4% (95% CI 2.6%–4.3%) of the patients after empirical prophylaxis and 0.8% after culture-based prophylaxis (95% CI 0.4%–1.3%).^10^ The other meta-analysis from nine cohort studies (4571 patients) also reported significantly higher post-biopsy infection rates after empirical prophylaxis (4.55%; 95% CI 3.80%–5.44%) compared with culture-based prophylaxis (0.72%; 95% CI 0.44%–1.18%).^3^

Innovative culture-based prophylaxis starts with pre-biopsy screening for FQ-resistant bacteria in the rectum of patients undergoing transrectal PB.^3^^,^^10^^,^^12^ Ideally, the swab is collected within 2 weeks before PB. When the swab is collected by the patient, this can be done either in the hospital or at home. Next, a bacterial culture test is performed at the clinical microbiological laboratory to identify men with FQ-resistant rectal Gram-negative bacteria where FQ prophylaxis is not appropriate. Moreover, the suitability of other antibiotics is assessed by bacterial culture tests and/or antibiotic susceptibility testing. Finally, urologists prescribe antibiotic prophylaxis based on these culture results.

Culture-based prophylaxis strategies require changes in the routine practices of healthcare providers and the usual care for patients involved. The acceptability of this strategy affects its uptake in daily clinical practice.^13^ The aim of this study was to explore experiences of healthcare providers and patients with culture-based oral antibiotic prophylaxis in transrectal PB following the patient care pathway (i.e. from the choice of culture-based prophylaxis to the ultimate prescription of culture-based antibiotic prophylaxis with a focus on the collection and processing of swabs).

## Patients and methods

This study on experiences was part of the PRO-SWAP trial (ClinicalTrials.gov; NCT03228108) (see below). We conducted focus group interviews with participating urologists and medical microbiologists to explore the barriers and facilitators they experienced regarding culture-based prophylaxis in transrectal PB. Moreover, the experiences of laboratory technicians and patients were investigated by means of questionnaires.

### PRO-SWAP trial details

The PRO-SWAP trial is a prospective non-blinded randomized controlled trial that aims to assess the effectiveness and cost-effectiveness of culture-based antibiotic prophylaxis to reduce infectious complications after transrectal PB. The trial started in April 2018 in three Dutch hospitals and two connected Dutch clinical microbiological laboratories and was expanded to 11 Dutch hospitals and six connected Dutch clinical microbiological laboratories during the trial. Patients were randomized into two groups of 666 patients each: a control group receiving routine empirical prophylaxis with oral ciprofloxacin 500 mg 2 h before and 12 h after prostate biopsy and an intervention group receiving rectal culture-based oral antibiotic prophylaxis. The antibiotics used in the intervention group are ciprofloxacin, trimethoprim/sulfamethoxazole, fosfomycin or pivmecillinam/amoxicillin/clavulanic acid. The primary outcome measure was any registered clinical infectious complication within 7 days after transrectal PB.

### Focus group interviews among urologists and microbiologists

Urologists and microbiologists involved in our trial were invited to participate in focus group interviews of 30–45 minutes each. Recruitment of urologists took place in 11 Dutch hospitals: one university hospital, eight non-university teaching hospitals and two non-university non-teaching hospitals. Medical microbiologists were recruited from the six connected Dutch clinical microbiological laboratories.

Healthcare providers were informed by e-mail about the research objectives, and subsequently invited to participate. Primarily, we scheduled face-to-face meetings for the focus groups (May and June 2020), but due to the COVID-19 pandemic we switched to video conferencing using the Zaurus application (a secured digital communication tool for healthcare consultations) (Zaurus, Alkmaar, the Netherlands). We aimed for groups of four to eight participants.

Focus group interviews were led by an experienced moderator (A.M.P.H.) in the presence of one researcher (S.C.M.T.). We used a semi-structured topic guide (Supplement S1; available as Supplementary data at *JAC-AMR* Online) based on a checklist for identifying determinants (barriers or facilitators) of practice, synthesized by Flottorp *et al.*^14^ We discussed determinants while following the various steps in the patientcare pathway.

### Questionnaire study among laboratory technicians

Laboratory technicians’ perceptions can differ from those of medical microbiologists because they actually perform the bacterial culture in daily clinical practice and can encounter other (mainly logistic) barriers. Therefore, while adapting to the COVID-19 pandemic and building on the information from the interviews, we developed a short online questionnaire to assess barriers and facilitators experienced while performing the cultures. The questionnaire was sent to 10 laboratory technicians who have experience with the culture-based prophylaxis strategy in the context of our PRO-SWAP trial. They were recruited from the same laboratories as the medical microbiologists. Open-ended questions explored the presence of any barrier (Supplement S2).

### Questionnaire study among patients

To develop a questionnaire, we searched MEDLINE^®^ for literature on patient experiences with self-sampling, published between January 2003 and November 2018 (see Supplement S3 for search terms, eligibility criteria and screening procedure). Topics were extracted from selected papers and categorized according to the Acceptability Framework by Sekhon *et al.*^13^ We used the qualitative software program ATLAS.ti (version 8.4.20) to accommodate this process. Topics were translated into items to measure experiences with self-sampling or sampling by healthcare providers.

In our PRO-SWAP trial, hospitals were allowed to determine the screening scenario themselves. We developed three questionnaires in line with the three potential swab screening scenarios: self-sampling at home, self-sampling in the hospital, and sampling by a healthcare provider in the hospital (Supplement S4). The questionnaire asked about experiences before, during and after collection of the swab. Questionnaires fitting the patient’s actual scenario were sent after rectal swab collection, but prior to PB to 452 patients from nine hospitals between 24 July 2019 and 9 March 2020. Patients were given the option to receive the questionnaire by e-mail or post. Patient inclusion was stopped at 9 March 2020 when the first national measures regarding the COVID-19 pandemic were announced by the Dutch government as the pandemic experience might affect patient experiences.

### Data analysis

The focus groups interviews were recorded and transcribed verbatim by an independent transcriber while maintaining the anonymity of the participants. The transcripts were analysed using a thematic content analysis approach based on the seven domains of Flottorp *et al.*^14^ with ATLAS.ti (version 8.4.20). To increase intercoder reliability, all transcripts were independently coded by the moderator (A.M.P.H.) and researcher (S.C.M.T.). Any discrepancies in the analysis were discussed until consensus was reached. Thematic analysis was also used for the open-ended questions among laboratory technicians.

Descriptive statistical analysis was performed for the questionnaires (patients and laboratory technicians) using SPSS version 25.0 (IBM Corp., Armonk, NY, USA). Both completed and partially completed questionnaires were analysed using the number of completed responses per item as the denominator.

Ethics approval was obtained from the regional medical ethics committee (NL63566.091.17). After healthcare providers were informed about the research objectives, data collection and analysis, and the procedures to maintain confidentiality of the research data, verbal informed consent was obtained from all healthcare providers prior to the focus group. Informed consent of the laboratory technicians was implied by completing and sending the questionnaire. All patients signed written consent forms before participating.

## Results

### Study population

We scheduled two focus group interviews with urologists. Eight urologists from eight different hospitals agreed to participate of whom four were present in one focus group; the second focus group interview was converted into an individual interview, because three urologists had to cancel the focus group at the last minute.

In addition, we conducted one focus group interview of approximately 40 minutes with medical microbiologists in which five of the seven intended participants from five different laboratories were present.

Eight laboratory technicians (80%) from five different laboratories returned the questionnaire. A total of 430 (95.1%) patients from nine different Dutch hospitals returned the questionnaire. Patient characteristics categorized by swab screening scenario i.e. self-sampling at home (40 patients), self-sampling in the hospital (345 patients) or sampling by a healthcare provider in the hospital (45 patients) are listed in Table 1.

**Table 1.** Patient characteristics

	Swab screening scenario		
	Self-sampling at home	Self-sampling in the hospital	Sampling by a healthcare provider
Patients, *n* (% of total, *n = *431)	40 (9.3%)	345 (80.2%)	45 (10.5%)
Number of hospitals, *n* (% of total, *n = *9)	8 (88.9%)	8 (88.9%)	5 (55.6%)
Division of patients per hospital, *n* (% within scenario group)			
Hospital A	1 (2.5%)	9 (2.6%)	1 (2.2%)
Hospital B	2 (5.0%)	20 (5.8%)	9 (20.0%)
Hospital C	4 (10.0%)	90 (26.1%)	11 (24.4%)
Hospital D	1 (2.5%)	1 (0.3%)	22 (48.9%)
Hospital E	8 (20.0%)	142 (41.2%)	2 (4.4%)
Hospital F	9 (22.5%)	2 (0.6%)	0 (0.0%)
Hospital G	1 (2.5%)	49 (14.2%)	0 (0.0%)
Hospital H	0 (0.0%)	32 (9.3%)	0 (0.0%)
Hospital I	14 (35.0%)	0 (0.0%)	0 (0.0%)
Patient age, years, median (IQR)	67 (63–72)	69 (64–73)	70 (62–75)
Highest educational attainment, *n* (%)			
Never finished school, primary school or elementary school	1 (2.5%)	31 (9.0%)	4 (8.9%)
Junior or intermediate vocational education or lower general secondary school	14 (35.0%)	158 (45.8%)	28 (62.2%)
Higher general secondary education or school for higher vocational education	19 (47.5%)	105 (30.4%)	10 (22.2%)
University	4 (10.0%)	38 (11.0%)	3 (6.7%)
Other	2 (5.0%)	11 (3.2%)	0 (0%)
Unknown	0 (0%)	2 (0.6%)	0 (0%)

Below, the results of all participants are collated and described along the various steps of the patientcare pathway.

### Choice for culture-based prophylaxis (urologists)

All urologists indicated that the effectiveness of the culture-based prophylaxis strategy in reducing post-biopsy infectious complications is decisive in their decision to implement the strategy in daily clinical practice: *‘is it really better than what we do now by default? Then—I think—it is very logical that we will implement it.*’ [Urologist 1; focus group]. Urologists stated that currently the quality of evidence is not sufficient, but that hopefully the PRO-SWAP trial will change this.

Moreover, urologists agreed that the culture-based prophylaxis strategy should preferably not affect the speed of prostate diagnostics or interfere with the fixed timeslots for certain diagnostic procedures along the total cancer diagnostic pathway since this is an important quality standard for them. Although urologists emphasized the tension between efficient oncology care pathways and the culture-based prophylaxis strategy, they stated: ‘*if the strategy is effective then our selling point that it goes fast, fast, fast becomes less important.’* [Urologist 5; interview]. Moreover, they believed that patients would understand that the diagnostic process takes slightly longer, so that culture-based prophylaxis can be given to reduce the risk of infectious complications.

### Choice of swab screening scenario (urologists, patients)

All urologists agreed that patients can easily collect the rectal swab themselves. Urologists assumed, however, that rectal swab collection within the hospital might be less error-prone than self-sampling at home: *‘the swab could be lost or not be returned. I would not argue in favour of giving the post a structural place in our diagnostic process.’* [Urologist 5; interview]. They suggested that rectal swab could be collected in the hospital toilet or in a separate room at the outpatient clinic, the latter of which may be more convenient for patients. In that case, healthcare assistants could also easily instruct patients. However, depending on the established logistic pathway around prostate cancer in a hospital, home-based self-sampling was considered a good option as well.

Opinions diverged slightly on whether the urologist could collect the swab during outpatient consultation. Some urologists indicated it was no problem for them to do so: *‘of course it takes a little more time, but I don’t think that’s a problem.’* [Urologist 4; focus group]. In contrast, another urologist stated: ‘*I calculate the risk of prostate cancer after rectal examination. Then I decide if a PB is necessary. But at that moment the patient is already dressed again. It is not practical to ask the patient to undress again in order to collect the swab.’* [Urologist 5; interview]. While another urologist noted: ‘*I think you should provide comfort to a patient who is already anxious about the presence of prostate cancer and let the rectal swab be collected by healthcare assistants at the outpatient clinic.’* [Urologist 2; focus group].

After rectal sampling, all patients were asked about their preference regarding who should collect the swab (self, healthcare provider, no preference) and where it should take place (hospital, home, no preference) (Table 2). Patients were quite satisfied with the care they received. In the group of patients where the swab was collected by the healthcare provider, <5% of all patients preferred self-sampling. In the group of patients who self-sampled, about 15% would have preferred sampling by a healthcare provider (14.7% of the patients with higher general secondary education or more; 22.2% of the patients with intermediate vocational education or less). In all three scenario groups, approximately one in three patients had no preference regarding who should collect the swab. Similar results were found with regard to sample collection location.

**Table 2.** Patient preferences

Question	Swab screening scenario		
	Self-sampling at home	Self-sampling in the hospital	Sampling by a healthcare provider
*If you had the choice: would you rather collect the swab yourself or have it collected by a doctor or nurse?*			
Self, *n* (%)	18 (46.2%)	181 (54.0%)	2 (4.4%)
By doctor or nurse, *n* (%)	7 (17.9%)	44 (13.1%)	29 (64.4%)
I have no preference, *n* (%)	14 (35.9%)	110 (32.8%)	14 (31.1%)
*If you had the choice: would you rather collect the swab at home or in the hospital?*			
At home, *n* (%)	21 (53.8%)	56 (16.7%)	3 (6.7%)
In the hospital, *n* (%)	4 (10.3%)	96 (28.7%)	23 (51.1%)
I have no preference, *n* (%)	14 (35.9%)	183 (54.6%)	19 (42.2%)
Total (*n*)	39	335	45

### Patients’ experiences before sampling

Results of the questionnaires for the three swab-screening scenarios can be found in Table 3. Free-text comments of patients can be found in Supplement S5.

**Table 3.** Results of the patient questionnaires for the three swab screening scenarios

Question	Scenario	Disagree + strongly disagree	Slightly disagree	Slightly agree	Agree + Strongly agree	I don’t know; NA
*Before collection of the swab*						

I found the explanation on the instruction form clear	1. Patient home (37)	2.7%	0.0%	10.8%	86.5%	0.0%
	2. Patient hospital (334)	3.3%	1.8%	2.4%	90.4%	2.1%

I found the explanation I received prior to collection of the swab clear	3. HP hospital (37)	5.4%	2.7%	5.4%	81.1%	5.4%

I was afraid that I was physically incapable to collect the swab properly	1. Patient home (40)	92.5%	0.00%	2.5%	5.0%	0.0%
	2. Patient hospital (342)	86.0%	2.9%	5.6%	3.2%	2.3%
	3. HP hospitala (4)	75.0%	25.0%	0.0%	0.0%	0.0%

I was confident that I could collect the swab properly	1. Patient home (40)	10.0%	2.5%	5.0%	82.5%	0.0%
	2. Patient hospital (341)	4.7%	3.5%	5.0%	84.8%	2.1%
	3. HP hospitala (4)	25.0%	50.0%	0.0%	25.0%	0.0%

I was confident that the doctor/nurse was capable to collect the swab properly	1. Patient home	NA	NA	NA	NA	NA
	2. Patient hospital	NA	NA	NA	NA	NA
	3. HP hospital (37)	0.0%	0.0%	2.7%	91.9%	5.4%

I was afraid to hurt myself when collecting the swab	1. Patient home (37)	89.2%	2.7%	5.4%	2.7%	0.0%
	2. Patient hospital (331)	87.0%	2.1%	4.8%	3.6%	2.4%

I was afraid that collecting the swab would hurt	3. HP hospital (37)	78.4%	2.7%	8.1%	8.1%	2.7%

I appreciated the opportunity to collect the swab myself	1. Patient home (37)	5.4%	0.0%	0.0%	89.2%	5.4%
	2. Patient hospital (329)	7.6%	3.7%	6.4%	73.0%	9.4%
	3. HP hospital	NA	NA	NA	NA	NA

I appreciated being able to decide when to collect the swab	1. Patient home (36)	5.6%	0.0%	0.0%	80.6%	13.9%
	2. Patient hospital	NA	NA	NA	NA	NA
	3. HP hospital	NA	NA	NA	NA	NA

I was afraid that it would be difficult to collect the swab	1. Patient home (40)	77.5%	2.5%	12.5%	7.5%	0.0%
	2. Patient hospital (337)	80.7%	4.8%	5.9%	6.5%	2.1%
	3. HP hospitala (4)	50.0%	0.0%	25.0%	25.0%	0.0%

I was afraid I couldn’t collect the swab on my own	1. Patient home (40)	82.5%	0.0%	5.0%	12.5%	0.0%
	2. Patient hospital (337)	84.3%	5.0%	4.2%	4.5%	2.1%
	3. HP hospitala (4)	75.0%	0.0%	0.0%	25.0%	0.0%

*During collection of the swab*						

I was able to collect the swab on my own	1. Patient home (37)	0.0%	0.0%	0.0%	97.3%	2.7%
	2. Patient hospital (330)	5.5%	1.5%	2.1%	89.7%	1.2%
	3. HP hospital	NA	NA	NA	NA	NA

I felt that, if needed, I could ask for help with the collection of the swab	1. Patient home (37)	24.3%	2.7%	5.4%	48.7%	18.9%
	2. Patient hospital (327)	28.1%	4.3%	4.3%	48.3%	15.0%
	3. HP hospital	NA	NA	NA	NA	NA

I was physically capable to collect the swab properly	1. Patient home (37)	2.7%	2.7%	0.0%	91.9%	2.7%
	2. Patient hospital (326)	2.5%	1.2%	3.1%	92.0%	1.2%
	3. HP hospital	NA	NA	NA	NA	NA

I found it difficult to collect the swab	1. Patient home (37)	86.5%	2.7%	2.7%	5.4%	2.7%
	2. Patient hospital (327)	81.7%	2.5%	7.0%	6.4%	2.5%
	3. HP hospital	NA	NA	NA	NA	NA
I found it annoying to touch myself in the area of the anus and intestine	1. Patient home (37)	81.1%	2.7%	5.4%	8.1%	2.7%
	2. Patient hospital (326)	86.5%	2.5%	5.2%	3.1%	2.8%
	3. HP hospital	NA	NA	NA	NA	NA

I found it difficult to insert the cotton swab	1. Patient home (37)	67.6%	8.1%	16.2%	5.4%	2.7%
	2. Patient hospital (326)	76.1%	4.9%	9.5%	7.1%	2.5%
	3. HP hospital	NA	NA	NA	NA	NA

I wasn’t sure if I had inserted the cotton swab deep enough	1. Patient home (37)	48.7%	10.8%	24.3%	13.5%	2.7%
	2. Patient hospital (324)	55.6%	5.6%	17.0%	17.9%	4.0%
	3. HP hospital	NA	NA	NA	NA	NA

I was afraid that the cotton swab might break while collecting the swab	1. Patient home (37)	81.1%	2.7%	10.8%	2.7%	2.7%
	2. Patient hospital (326)	86.5%	3.1%	4.0%	4.0%	2.5%

I was afraid that the cotton swab might break during the swab collection	3. HP hospital (41)	80.5%	0.0%	0.0%	7.3%	12.2%

I experienced pain while collecting the swab	1. Patient home (40)	80.0%	5.0%	7.5%	5.0%	2.5%
	2. Patient hospital (334)	86.8%	2.1%	6.9%	2.4%	1.8%

I experienced pain while the swab was collected	3. HP hospital (41)	85.4%	2.4%	0.0%	9.8%	2.4%

I felt shame while collecting the swab	1. Patient home (40)	87.5%	2.5%	0.0%	5.0%	5.0%
	2. Patient hospital (334)	94.0%	1.5%	1.2%	0.9%	2.4%

I felt shame while the swab was collected	3. HP hospital (41)	85.4%	0.0%	4.9%	7.3%	2.4%

I found it unsanitary to collect the swab myself	1. Patient home (37)	86.5%	2.7%	5.4%	2.7%	2.7%
	2. Patient hospital (325)	91.1%	1.9%	0.6%	3.7%	2.8%
	3. HP hospital	NA	NA	NA	NA	NA

I felt enough privacy while the swab was collected	1. Patient home	NA	NA	NA	NA	NA
	2. Patient hospital (334)	9.6%	1.8%	1.5%	84.1%	3.0%
	3. HP hospital (41)	2.4%	2.4%	2.4%	87.8%	4.9%

I am confident that I have collected the swab properly	1. Patient home (37)	2.7%	2.7%	8.1%	83.8%	2.7%
	2. Patient hospital (325)	4.9%	2.8%	8.9%	80.3%	3.1%

I am confident that the swab has been properly collected	3. HP hospital (36)	2.8%	2.8%	0.0%	91.7%	2.8%

*After collection of the swab*						

I was concerned that the cotton swab would contact unsanitary surfaces before it was placed into the tube	1. Patient home (37)	86.5%	0.0%	13.5%	0.0%	0.0%
	2. Patient hospital (324)	82.7%	2.5%	8.3%	4.3%	2.2%
	3. HP hospital	NA	NA	NA	NA	NA

I was concerned I would spill liquid from the tube	1. Patient home (37)	73.0%	2.7%	21.6%	2.7%	0.0%
	2. Patient hospital (324)	83.6%	0.9%	8.3%	4.9%	2.2%
	3. HP hospital	NA	NA	NA	NA	NA

I found it difficult to break the cotton stick in the tube	1. Patient home (37)	86.5%	5.4%	5.4%	2.7%	0.0%
	2. Patient hospital (324)	88.9%	2.5%	4.0%	2.8%	1.9%
	3. HP hospital	NA	NA	NA	NA	NA

I found it annoying to keep the tube in the refrigerator after sampling	1. Patient home (36)	72.2%	2.8%	0.0%	5.6%	19.4%
	2. Patient hospital	NA	NA	NA	NA	NA
	3. HP hospital	NA	NA	NA	NA	NA
It was clear how the tube should be packed in the special transport system	1. Patient home (37)	18.9%	10.8%	2.7%	67.6%	0.0%

It was clear how the tube should be packed	2. Patient hospital (324)	5.9%	0.6%	1.9%	89.8%	1.9%
	3. HP hospital	NA	NA	NA	NA	NA

It was clear where the tube should be hand in	1. Patient home	NA	NA	NA	NA	NA
	2. Patient hospital (324)	7.4%	2.8%	3.4%	85.2%	1.2%
	3. HP hospital	NA	NA	NA	NA	NA

I was afraid the package would get damaged or lost in the mail	1. Patient home (36)	66.7%	2.8%	11.1%	2.8%	16.7%
	2. Patient hospital	NA	NA	NA	NA	NA
	3. HP hospital	NA	NA	NA	NA	NA

I was concerned about the result of the test	1. Patient home (40)	70.0%	5.0%	10.0%	12.5%	2.5%
	2. Patient hospital (332)	62.7%	4.8%	12.7%	13.6%	6.3%
	3. HP hospital (41)	51.2%	4.9%	22.0%	17.1%	4.9%

HP, healthcare provider; NA, not applicable.

a Question was only asked to four patients (self-sampling scenario) who indicated that the swab was collected by the healthcare provider at the patient’s request.

In general, patients were positive about instructions. Self-sampling patients who received written information were slightly more positive (home: 86.5%; hospital: 90.4%) than patients who were verbally informed and sampled by a healthcare provider (81.1%).

More than 80% of the self-sampling patients were confident to be able to collect the swab on their own, while 91.9% of the patients where a healthcare provider collected the swab were confident that the doctor/nurse was capable of collecting the swab properly. About 8% of self-sampling patients were (somewhat) concerned that collecting the swab would hurt as compared with about 16% in the group where the healthcare provider collected the swab. Of the patients who self-sampled at home or in the hospital, 33 patients (89.2%) and 240 patients (73.0%) respectively, liked being given the opportunity to self-sample.

### Patients’ experiences during sampling

Again, for most topics, 80% or more of the patients had positive experiences. Among those patients who self-sampled, however, more than 15% (somewhat) experienced difficulties to insert the cotton swab (home: 21.6% and hospital 16.6%). These patients mainly found it difficult to find the right place of insertion or to get through the sphincter because the anus was dry. About 15%–20% of the patients who took self-samples was unsure whether they had inserted the cotton swab deep enough. Some patients were uncertain about this because there was no stool visible on the swab or because the swab was collected after an internal examination in which lubricant was used. Approximately 1 in 4 patients who collected the swab themselves, felt that, if desired, they could not ask for help to collect the swab. Some patients (free-text comments) reported that they asked their partner for help in collecting the swab. In general, patients did not find it unsanitary to collect the swab themselves.

In patients where the swab was collected by a healthcare provider more patients were afraid that the swab might break during sampling (3; 7.3%) as compared with the patients who self-sampled at home (1; 2.7%) or in the hospital (13; 4.0%). The same applies to the experience of pain and shame while collecting the swab.

Privacy was especially noted as a problem by patients who collected the swab in the hospital (9.6%). In the free-text comments, 20 patients reported about the discomfort of collecting the swab in the hospital toilet. They noted that they lacked a hygienic spot in the toilet room to deposit the swab materials and experienced insufficient room to collect the swab. Moreover, they indicated preference for a toilet room in a quiet environment, so not in the central hall of the hospital.

### Patients’ experiences after sampling

In the group of patients who self-sampled at home, patients clearly experienced difficulties in packaging the swab in the special transport system, since only 25 patients (67.6%) indicated this was clear. This percentage was much higher in patients who self-sampled in the hospital and had to pack the swab differently (89.8%), however, for 24 patients (7.4%) it was unclear where to hand in the swab. Approximately 1 in 8 patients in the self-sampling groups were concerned about the result of the culture test, compared with 1 in 6 patients in whom the swab was collected by a healthcare provider.

### Rectal culture processing at the clinical microbiological laboratory (medical microbiologists, laboratory technicians)

None of the medical microbiologists anticipated problems in applying bacterial culture methods to identify resistant Gram-negative bacteria prior to PB. This was supported by the laboratory technicians who summarized that their laboratory would support the task to process the rectal culture. Laboratory technicians stated they required a short training session of about 20 minutes, mainly regarding instruction on the protocol. They would appreciate the availability of a clear point of contact in case of problems or questions. In addition, laboratory technicians indicated that checking the shelf life of the culture media requires extra attention due to different expiration dates of the various media.

Medical microbiologists and laboratory technicians expected a minimal change in workload following the introduction of a culture-based prophylaxis strategy. Laboratory technicians anticipated that the culture process would take about 30 minutes.

When asked about the costs of the cultured-based prophylaxis strategy, medical microbiologists stated that *‘the culture process costs some money, but if it leads to quality gains within the hospital organization and if it really yields a profit for the patient, then the costs are fractional compared with what can be gained from it’*. [Medical microbiologist 3].

### Availability of results and prescription of culture-based antibiotic prophylaxis (urologists)

None of the urologists anticipated problems in relation to the timely assessment of rectal culture results and the subsequent prescription of culture-based antibiotic prophylaxis before PB. In all hospitals, logistics are already established: *‘the healthcare assistant ensures that a designated urologist receives an overview of all new culture results every day. Based on these culture results urologists can prescribe antibiotics, after which the healthcare assistant can contact the patient to indicate that the antibiotics are available at the pharmacy.’* [Urologist 5; interview].

## Discussion

**Figure 1.**
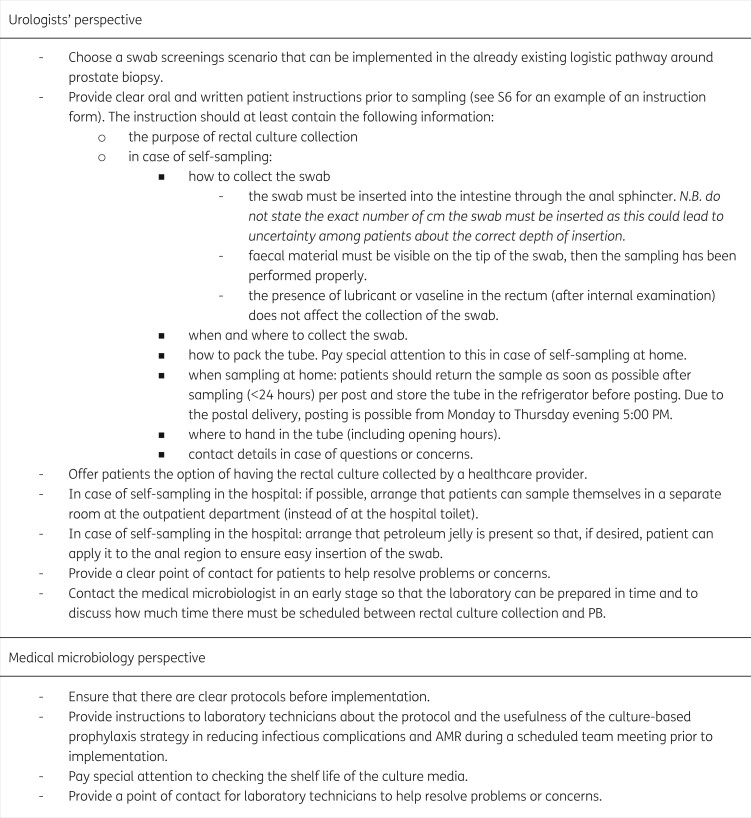
Tips and tricks for implementation of the culture-based prophylaxis strategy.

In this multi-method study, we explored the acceptability of culture-based antibiotic prophylaxis strategies in transrectal PB, based on the experiences and preferences of healthcare providers and patients. Overall, culture-based prophylaxis strategies were experienced as feasible in daily clinical practice and seem fairly easy to implement. Based on our results, we summarized tips and tricks to implement this strategy in daily clinical practice (Figure 1). A template for written instruction regarding the rectal swab collection can be found in Supplement S6. In our study, none of the three potential swab-screening scenarios performed better. For urologists, the choice for a particular screening scenario mainly depended on the simplicity with which rectal culture collection can be implemented in the existing logistic pathway around PB. A decisive factor for urologists would be that the strategy has no or limited influence on the speed of the cancer diagnostic process. Provided that these conditions are met, multiple scenarios within a hospital are probably needed to meet the various patients’ needs and preferences.

Patients were generally satisfied with the screening scenario presented to them regardless of which scenario it was. Self-samplers reported lower levels of shame and pain compared with patients who were sampled by a healthcare provider. However, trust in the correct execution of sampling was higher when a healthcare provider performed the rectal culture. These findings are in line with other qualitative studies addressing patients’ perspectives on self-sampling, e.g. with regard to human papillomavirus.^15^^,^^16^ Furthermore, in our study, about 15% of the self-samplers indicated that they would have preferred sampling by a healthcare provider (home: 17.9%; hospital: 13.1%). In addition, some self-samplers felt that they were not able or found it difficult to sample themselves, while 1 in 4 patients noted that, if needed, they could not ask for help. Based on these results, patients should at least be offered the option of having the rectal culture collected by a healthcare provider.

In our PRO-SWAP trial, hospitals were allowed to determine the screening scenario themselves. Since, the majority of the swabs were collected by patients in the hospital (80.2%), in our study, most urologists considered this strategy as the most practical method. In practice, often two or more scenarios were performed within one and the same hospital. It should be noted, however, that because our study was performed under the umbrella of a randomized controlled trial, preferences for the different screening scenarios are partly distorted by unequal inclusion rates between the different hospitals within our PRO-SWAP trial (see Table 1).

Urologists assumed that rectal swab collection within the hospital is less error-prone than self-sampling at home. Although no research has been performed about the diagnostic accuracy of rectal self-sampling for the purpose of culture-based prophylaxis, the accuracy of rectal self-sampling has been shown for various other purposes (group B streptococcus, chlamydia, gonorrhoea, human papillomavirus).^17–19^ In addition, a control was built into the laboratory diagnostics.

The acceptability of culture-based prophylaxis strategies and the success of implementation is highly dependent on the support base for the strategy. Urologists indicated that the support base is mainly related to the effectiveness of the culture-based prophylaxis strategy. Currently, the effectiveness of culture-based prophylaxis in PB has been shown mainly in (meta-analyses of) retrospective and consecutive prospective cohort studies.^3^ Our PRO-SWAP trial, focusing on oral alternative antibiotic prophylaxis in case of ciprofloxacin-resistant rectal flora, expected to be completed in mid-2021, will add to the evidence on this subject.

The main strength of our study is that we performed a structured multi-method analysis of the culture-based prophylaxis strategy among all stakeholders. It should, however, be taken into account that our study was conducted among healthcare providers and patients who had experience with culture-based antibiotic prophylaxis within a research context (PRO-SWAP trial). It cannot be ruled out that experiences are partly influenced by this e.g. the selection of healthcare providers from our PRO-SWAP trial may have influenced the responses, because of their (presumably) positive attitude towards the approach. Given that our study was conducted within a randomized controlled trial, and in one country, the generalizability of the results may be a concern. However, because the culture-based prophylaxis strategy is hardly used in daily clinical practice yet, it was not possible to perform our study in a non-research setting. In addition, all tools used in our study have been added as appendix to facilitate researchers/professionals in assessing the acceptability in other settings or countries. Second, it is important to note that we only explored the acceptability of oral culture-based antibiotic prophylaxis strategies. As a result, we cannot make statements about any barriers that may be experienced when testing for and administering intravenous antibiotics. Last, we experienced some limitations due to the COVID-19 pandemic which forced us to stop the patient questionnaire study earlier than anticipated and to switch from face-to-face focus group interviews to video conferencing.

This study has increased our understanding of the acceptability of oral culture-based antibiotic prophylaxis strategies in PB. If healthcare providers, based on its effectiveness, aim to implement the strategy, this information can contribute to successful implementation and high-quality patient-centred care.

## Supplementary Material

dlab161_Supplementary_Data
