# Supplementary material for: Acceptability of innovative culture-based antibiotic prophylaxis strategies: a multi-method study on experiences regarding transrectal prostate biopsy
Source: JAC Antimicrob Resist. 2021 Nov 17;3(4):dlab161. doi: 10.1093/jacamr/dlab161 (PMC8599774; doi:10.1093/jacamr/dlab161)
Supplement: dlab161_Supplementary_Data [file dlab161_supplementary_data.docx]

**Supplementary data**

**S1 - Topic guides for the focus groups**

**Topic guide for the focus groups with urologists**

*Purpose:*

To explore experiences, barriers, and facilitators regarding culture-based antimicrobial prophylaxis in transrectal prostate biopsy. Additionally, to identify solutions that facilitate future implementation of culture-based antimicrobial prophylaxis in daily clinical practice.

We used a semi-structured topic guide based on a checklist for identifying determinants (barriers or facilitators) of practice, synthesized by Flottorp *et al.* This checklist includes determinants grouped into seven domains: guideline factors; individual health professional factors; patient factors; professional interactions; incentives and resources; capacity for organizational change; and social, political and legal factors.

*Central questions:*

1. What barriers and facilitators do you encounter in performing a rectal culture to screen for resistant gram-negative bacteria prior to prostate biopsy? Please, keep in mind the following swab screening scenarios: self-sampling at home; self-sampling in the hospital; sampling by a healthcare provider in the hospital.
2. What barriers and facilitators do you encounter in prescribing antimicrobial prophylaxis prior to prostate biopsy based on rectal culture results?
3. What solutions are needed to address identified barriers?

*Points of interest*

GUIDELINE FACTORS - Strength of evidence / Feasibility and compatibility of culture-based antimicrobial prophylaxis within current logistical pathways for prostate diagnostics

INDIVIDUAL HEALTH PROFESSIONAL FACTORS – Awareness, knowledge, and skills of urologists, nurses, and other involved healthcare workers

PATIENT FACTORS – Knowledge and skills, beliefs, preferences, motivation, behaviour, (information) needs

INCENTIVES AND RESOURCES - Availability of necessary resources/ Financial incentives and disincentives

CAPACITY FOR ORGANISATIONAL CHANGE - Priority of necessary change/ Regulations, rules, and policies

All participants received a small fee for their participation in the form of a gift card.

**Topic guide for the focus groups with medical microbiologists**

*Purpose:*

To explore experiences, barriers and facilitators regarding culture-based antimicrobial prophylaxis in transrectal prostate biopsy. Additionally, to identify solutions that facilitate future implementation of culture-based antimicrobial prophylaxis in daily clinical practice.

We used a semi-structured topic guide based on a checklist for identifying determinants (barriers or facilitators) of practice, synthesized by Flottorp *et al.* This checklist includes determinants grouped into seven domains: guideline factors; individual health professional factors; patient factors; professional interactions; incentives and resources; capacity for organizational change; and social, political and legal factors.

*Central questions:*

1. What barriers and facilitators do you encounter in applying bacterial culture methods to identify resistant gram-negative bacteria prior to prostate biopsy?
2. What solutions are needed to address identified barriers?

*Points of interest:*

GUIDELINE FACTORS - Strength of evidence/ Feasibility and compatibility of logistical procedures in the laboratory (e.g. large scale testing)

INDIVIDUAL HEALTH PROFESSIONAL FACTORS – Awareness, knowledge, and skills among laboratory analysts

INCENTIVES AND RESOURCES - Availability of necessary resources/ Financial incentives and disincentives

CAPACITY FOR ORGANISATIONAL CHANGE - Priority of necessary change/ Regulations, rules, and policies

**S2 – Questionnaire for laboratory analysts**

Dear analyst,

As you know, the PRO-SWAP study is currently running in more than 10 Dutch hospitals: a study in patients undergoing a prostate biopsy.

Patients who undergo a prostate biopsy in the Netherlands are given antibiotics (usually ciprofloxacin) around the procedure. These prophylactic antibiotics should prevent post-biopsy infectious complications. In the PRO-SWAP study, we investigate if patients are better protected against infections if a rectal swab is collected prior to prostate biopsy. In this rectal swab, we test whether there is resistance to ciprofloxacin or three other oral antibiotics that can be a prophylactic alternative. To this end, we have developed selective agars on which the material of the rectal swab can be cultured.

If our study shows that this so-called culture-based prophylaxis strategy results in fewer infections, the PRO-SWAP research group will advise the relevant guideline committees to recommend culture-based prophylaxis. To facilitate any future implementation of culture-based prophylaxis, we also intend to make recommendation **on how to implement the culture-based approach in daily clinical practice.**

As analyst, you are the person who is on the work floor performing the bacterial culture tests. We would like to hear your experiences with this so that hospitals that do not yet work with this strategy can learn from you. We would therefore appreciate it if you are willing to answer a number of questions.

Thank you very much in advance,

Sincerely,

Sofie Tops, PhD student PRO-SWAP

Prof. dr. Heiman Wertheim, clinical microbiologist Radboudumc

Dr. Eva Kolwijck, clinical microbiologist Radboudumc

Prof. dr. Marlies Hulscher, IQ Healthcare

Dr. Anita Huis, IQ Healthcare

**Questionnaire**

**Important: Please read this first!**

We ask you to assume that the culture-based prophylaxis strategy is superior to the current prophylaxis for prostate biopsy in which everyone receives standard ciprofloxacin. The updated guidelines therefore recommend to collect a rectal swab prior to prostate biopsy in order to identify resistant gram-negative bacteria. In the Netherlands, this concerns 300-500 patients per hospital on an annual basis.

According to the current laboratory protocol of the study, this would mean for an average Dutch hospital laboratory that 1 drop of the Eswab medium must be placed and spatulated on four selective agars with antibiotics 300-500 times a year. All agars should be evaluated for growth after 24 and 48 hours. If there is growth, a MALDI-TOF should be performed from each unique morphological colony derived from the selective agars. In case of growth on all four selective agars, antimicrobial susceptibility tests must be performed of each unique morphological colony on the selective plate with ciprofloxacin by means of E-test strips for five different antibiotics. The latter is the case in about 1 in 10 cultures (so 30-50 times on an annual basis).

**Imagine: your colleague from a similar hospital that did not participate in the PRO-SWAP study is asking you for help in shaping this new activity in their laboratory. He/she does not want to reinvent the wheel himself/herself and asks you to indicate in detail what problems you encountered while carrying out the above steps, whether you solved them and if so: how? The colleague is interested in any problem, large and small: shortages in knowledge or skills, problems in collaboration, coordination or communication with colleagues in the lab, lack of motivation, problems with finances, lack of materials in stock or other logistical problems, unclear results, problems with the execution in the weekend etc etc.**

**YOUR COLLEAGUE ASKS YOU ABOUT THE PROBLEMS YOU HAVE ENCOUNTERED AND WHETHER YOU HAVE SOLVED THEM IN YOUR HOSPITAL.**

*Fill in the questions based on your experience and your opinion. There are no right or wrong answers.*

**How long does it take to complete the questionnaire?**

It takes a maximum of 15 minutes to complete the questionnaire.

**What will happen with your answers?**

Your answers will be used for research. Only the researchers will see your answers. So no one else. The researchers will not tell anyone that you cooperated.

**Thank you for completing the questionnaire for us!**

**Please fill in the most appropriate answer and, where requested, give a description as specific as possible?**

*Culture process*

- 1. When receiving/registering the culture
- no obstacles were experienced
- obstacles were experienced, namely:

**Problem 1:**

This problem:

- is not solved (yet)
- was solved as follows:

Did you experience any more obstacles when receiving/registering the culture:

- no
- yes, namely:

**Problem 2: (if yes)**

This problem:

- is not solved (yet)
- was solved as follows:

Did you experience any more obstacles when receiving/registering the culture:

- no
- yes, namely:

**Problem 3: (if yes)**

This problem:

- is not solved (yet)

was solved as follows

- 1. When processing the culture (inoculation, checking for bacterial growth and

identification of micro-organism by means of MALDI-TOF)

- no obstacles were experienced
- obstacles were experienced, namely:

*same step-by-step construction as above*

- 1. When interpretating the culture
- no obstacles were experienced
- obstacles were experienced, namely:

*Think about, for example, assessing the need for additional susceptibility testing.*

*same step-by-step construction as above*

- 1. When rapporting the culture
- no obstacles were experienced
- obstacles were experienced, namely:

*same step-by-step construction as above*

- 1. In the throughput time of the culture
- no obstacles were experienced
- obstacles were experienced, namely:

*Also think about the routing in the weekend.*

*same step-by-step construction as above*

*Culture agars*

- 1. During the ordering process of the agars
- no obstacles were experienced
- obstacles were experienced, namely:

*same step-by-step construction as above*

- 1. With the delivery time of the agars (maximum five working days)
- no obstacles were experienced
- obstacles were experienced, namely:

*same step-by-step construction as above*

- 1. During the internal quality control of the agars
- no obstacles were experienced
- obstacles were experienced, namely:

*same step-by-step construction as above*

- 1. With regard to the shelf life of the agars (10 weeks from production; in practice

a bit shorter)

- no obstacles were experienced
- obstacles were experienced, namely:

*same step-by-step construction as above*

*General questions*

Below are a number of questions. Would you please answer this as completely, accurately and concretely as possible?

1. The entire process from receipt of the culture to reporting the culture result takes

about ….. minutes per patient..

2.1 Training or instruction of the personnel for this new method requires approximately ….. minutes of time.

2.2 Personnel must be instructed/trained on the new working method, and in particular in……

2.3 Do you have any general tips that you would like to give your colleague from a similar hospital that did not participate in the PRO-SWAP study with regard to shaping the culture-based prophylaxis strategy in their laboratory?

*Consequences for your own laboratory*

If the PRO-SWAP study is finished and the culture-based prophylaxis strategy would be included in a new guideline, a number of things will also change in your laboratory, namely:

1. The number of rectal cultures you will receive will increase to 300-500 patients per hospital on an annual basis.
2. In case of growth on all four selective agars, you will need to perform additional susceptibility tests yourself. Concrete, this means that you must perform E-tests for five different antibiotics of each unique morphological colony derived from the selective agar with ciprofloxacin. The latter is the case in about 1 in 10 cultures (so 30-50 times on an annual basis).
3. Imagine: based on the increased number of rectal cultures your lab will receive,

it is decided to process the rectal cultures via an automated microbiology

specimen processing instrument or inoculate the selective agars automatically.

Hereby:

- I do not expect any obstacle to occur/I do not see any objections
- I do expect any obstacle to occur/I do see any objections, namely

*same step-by-step construction as above*

3.1. When performing additional susceptibility tests (in case of growth on all four selective agars with antibiotics)

- I do not expect any obstacle to occur/I do not see any objections
- I do expect any obstacle to occur/I do see any objections, namely

*Note: E-test strips for fosfomycin should be placed on a Mueller-Hinton agar with glucose-6-phosphate.*

*same step-by-step construction as above*

3.2 To what extent do you expect a change in workload because you have to process rectal cultures for 300-500 patients per hospital on an annual basis. Use a scale of 0-10, where 0 means “no change” and 10 means “very, very, much change”.

3.3. To what extent is there support/motivation in your laboratory for processing rectal cultures? Use a scale of 0-10, where 0 means “not at all” and 10 means “very, very, much”.

3.4 Which factors could increase the support/motivation for processing the rectal cultures in your laboratory?

Maybe you have any comments or questions. You can place these below.

**Thank you very much for completing this questionnaire!**

**S3 – Search terms for literature on patient experiences with self-sampling**

Studies were eligible if (i) they involved human participants who had undergone self-sampling; (ii) they reported on actual experiences of the participants (studies describing only uptake and/or willingness to undergo self-sampling were excluded); (iii) used the following self-obtained samples: vaginal/rectal/penile/vulval/oral/nasal swabs, cervical/vaginal lavage, cervical brush, tampons; and (iv) were published in English. Two reviewers independently screened titles and abstracts for articles that met the inclusion criteria. We used Rayyan, a systematic reviews web app, for this screening process. Articles selected for full text screening were independently assessed by one reviewer (WT). A second reviewer (MH) randomly and independently assessed half of the articles. Disagreements in selections and extracted data from the articles were resolved by consensus.

A total of 816 articles were identified from the computerized search; 647 were excluded after screening titles and abstracts. Thirty-eight papers explicitly set out to explore experiences using questionnaires or interviews; 131 papers briefly touched upon experiences, most often they used 1-5 items asking for example on acceptability, physical comfort, pain, or trust in safety of the test.

**Overview of search terms used**

("Specimen Handling/psychology"[Mesh] OR "Self-Examination/methods"[Mesh] OR Self-sampl*[tiab] OR Self-swab*[tiab] OR Home-sampl*[tiab] OR ((Self-test*[tiab] OR Home-test*[tiab] OR Self-collect*[tiab] OR Home-collect*[tiab]) AND (sampl*[tiab] OR swab*[tiab] OR smear[tiab] OR specimen[tiab] OR specimens[tiab])) OR (Home[tiab] AND swab*[tiab])) AND (“rectum” [Mesh] OR Rectal [tiab] OR Rectum [tiab] OR “Anal Canal” [Mesh] OR Anorectal [tiab] OR Anal [tiab] OR “Saliva” [Mesh] OR Saliva [tiab] OR Oral fluid [tiab] OR Salivary [tiab] OR Pharyngeal [tiab] OR Mucosa* [tiab] OR Mucus [tiab] OR mucosal secretions [tiab] OR oral [tiab] OR “cervix mucus” [Mesh] OR “vaginal smears/methods”[Mesh] OR “vaginal smears/psychology”[Mesh] OR “vaginal smears/statistics &numerical data”[Mesh] OR Cervicovaginal [tiab] OR vaginal [tiab] OR Cervical [tiab] OR “nasal mucosa”[Mesh] OR penile [tiab]OR Nasal [tiab] OR HPV [tiab] OR STI [tiab] OR HIV [tiab]) AND ("Patient Acceptance of Health Care"[Mesh] OR "Patient Satisfaction"[Mesh] OR Acceptab*[tiab] OR Acceptanc*[tiab] OR Prefer*[tiab] OR Experience*[tiab] OR Satisf*[tiab] OR Barrier*[tiab] OR Attitude*[tiab] OR concern [tiab] OR concerns [tiab] OR subjective [tiab])

**S4 – Patient questionnaires**

***Scenario 1:* self-sampling at home**

**Collection of the cotton swab**

Immediately after your appointment with the urologist in the hospital, you were asked to collect a rectal swab. The following questions are about your experiences with this procedure.

Information about your experiences helps us -if necessary- to improve the information and logistics regarding the rectal swab collection.

Below we will ask you about your experiences **before** sampling, **during** sampling and **after** sampling.

**Please indicate the situation that applies to you:**

- The swab was collected by a doctor or nurse on the initiative of the doctor or nurse
- The swab was collected by a doctor or nurse at my request
- I collected the swab myself on the toilet in the hospital
- I collected the swab myself at home
- Otherwise, namely

**If needed, you can explain your answer here:**

**Your preferences:**

1. If you had the choice: would you rather collect the swab yourself or have it collected by a doctor or nurse?

- Self
- By doctor or nurse
- I have no preference

1. If you had the choice: would you rather collect the swab at home or in the hospital?
   - At home
   - In the hospital
   - I have no preference

**Before collection of the swab**

|  | Strongly disagree | Disagree | Slightly dis-agree | Sligthly agree | Agree | Strongly agree | I don’t know; n/a |
| --- | --- | --- | --- | --- | --- | --- | --- |
| 3. I found the explanation on the instruction form clear |  |  |  |  |  |  |  |
| 4. I was afraid that I was physically incapable to collect the swab properly |  |  |  |  |  |  |  |
| 5. I was confident that I could collect the swab properly |  |  |  |  |  |  |  |
| 6. I was afraid to hurt myself when collecting the swab |  |  |  |  |  |  |  |
| 7. I appreciated the opportunity to collect the swab myself |  |  |  |  |  |  |  |
| 8. I appreciated being able to decide when to collect the swab |  |  |  |  |  |  |  |
| 9. I was afraid that it would be difficult to collect the swab |  |  |  |  |  |  |  |
| 10. I was afraid I couldn’t collect the swab on my own |  |  |  |  |  |  |  |

**Do you have any questions or comments? For example, did you miss or need certain things? If so, please write it down below.**

**During collection of the swab**

|  | Strongly disagree | Disagree | Slightly dis-agree | Sligthly agree | Agree | Strongly agree | I don’t know; n/a |
| --- | --- | --- | --- | --- | --- | --- | --- |
| 11. I was able to collect the swab on my own |  |  |  |  |  |  |  |
| 12. I felt that, if needed, I could ask for help with the collection of the swab |  |  |  |  |  |  |  |
| 13. I was physically capable to collect the swab properly |  |  |  |  |  |  |  |
| 14. I found it difficult to collect the swab |  |  |  |  |  |  |  |
| 15. I found it annoying to touch myself in the area of ​​the anus and intestine |  |  |  |  |  |  |  |
| 16. I found it difficult to insert the cotton swab |  |  |  |  |  |  |  |
| 17. I wasn’t sure if I had inserted the cotton swab deep enough |  |  |  |  |  |  |  |
| 18. I was afraid that the cotton swab might break the swab collection |  |  |  |  |  |  |  |
| 19. I experienced pain while collecting the swab |  |  |  |  |  |  |  |
| 20. I felt shame while collecting the swab |  |  |  |  |  |  |  |
| 21. I found it unsanitary to collect the swab myself |  |  |  |  |  |  |  |
| 22. I am confident that I have collected the swab properly |  |  |  |  |  |  |  |

**Do you have any questions or comments? For example, did you miss or need certain things? If so, please write it down below.**

**After collection of the swab**

|  | Strongly disagree | Disagree | Slightly dis-agree | Sligthly agree | Agree | Strongly agree | I don’t know; n/a |
| --- | --- | --- | --- | --- | --- | --- | --- |
| 23. I was concerned that the cotton swab would contact unsanitary surfaces before it was placed into the tube |  |  |  |  |  |  |  |
| 24. I was concerned I would spill liquid from the tube |  |  |  |  |  |  |  |
| 25. I found it difficult to break the cotton stick in the tube |  |  |  |  |  |  |  |
| 26. I found it annoying to keep the tube in the refrigerator after sampling |  |  |  |  |  |  |  |
| 27. It was clear how the tube should be packed in the special transport system |  |  |  |  |  |  |  |
| 28. I was afraid the package would get damaged or lost in the mail |  |  |  |  |  |  |  |
| 29. I was concerned about the result of the test |  |  |  |  |  |  |  |

**Do you have any questions or comments? For example, did you miss or need certain things? If so, please write it down below.**

***Scenario 2:* self-sampling in the hospital**

**Collection of the cotton swab**

Immediately after your appointment with the urologist in the hospital, you were asked to collect a rectal swab. The following questions are about your experiences with this procedure.

Information about your experiences helps us -if necessary- to improve the information and logistics regarding the rectal swab collection.

Below we will ask you about your experiences **before** sampling, **during** sampling and **after** sampling.

**Please indicate the situation that applies to you:**

- The swab was collected by a doctor or nurse on the initiative of the doctor or nurse
- The swab was collected by a doctor or nurse at my request
- I collected the swab myself on the toilet in the hospital
- I collected the swab myself at home
- Otherwise, namely

**If needed, you can explain your answer here:**

**Your preferences:**

1. If you had the choice: would you rather collect the swab yourself or have it collected by a doctor or nurse?

- Self
- By doctor or nurse
- I have no preference

1. If you had the choice: would you rather collect the swab at home or in the hospital?
   - At home
   - In the hospital
   - I have no preference

**Before collection of the swab**

|  | Strongly disagree | Disagree | Slightly dis-agree | Sligthly agree | Agree | Strongly agree | I don’t know; n/a |
| --- | --- | --- | --- | --- | --- | --- | --- |
| 3. I found the explanation on the instruction form clear |  |  |  |  |  |  |  |
| 4. I was afraid that I was physically incapable to collect the swab properly |  |  |  |  |  |  |  |
| 5. I was confident that I could collect the swab properly |  |  |  |  |  |  |  |
| 6. I was afraid to hurt myself when collecting the swab |  |  |  |  |  |  |  |
| 7. I appreciated the opportunity to collect the swab myself |  |  |  |  |  |  |  |
| 8. I was afraid that it would be difficult to collect the swab |  |  |  |  |  |  |  |
| 9. I was afraid I couldn’t collect the swab on my own |  |  |  |  |  |  |  |

**Do you have any questions or comments? For example, did you miss or need certain things? If so, please write it down below.**

**During collection of the swab**

|  | Strongly disagree | Disagree | Slightly dis-agree | Sligthly agree | Agree | Strongly agree | I don’t know; n/a |
| --- | --- | --- | --- | --- | --- | --- | --- |
| 10. I was able to collect the swab on my own |  |  |  |  |  |  |  |
| 11. I felt that, if needed, I could ask for help with the collection of the swab |  |  |  |  |  |  |  |
| 12. I was physically capable to collect the swab properly |  |  |  |  |  |  |  |
| 13. I found it difficult to collect the swab |  |  |  |  |  |  |  |
| 14. I found it annoying to touch myself in the area of ​​the anus and intestine |  |  |  |  |  |  |  |
| 15. I found it difficult to insert the cotton swab |  |  |  |  |  |  |  |
| 16. I wasn’t sure if I had inserted the cotton swab deep enough |  |  |  |  |  |  |  |
| 17. I was afraid that the cotton swab might break during the swab collection |  |  |  |  |  |  |  |
| 18. I experienced pain while collecting the swab |  |  |  |  |  |  |  |
| 19. I felt shame while collecting the swab |  |  |  |  |  |  |  |
| 20. I found it unsanitary to collect the swab myself |  |  |  |  |  |  |  |
| 21. I felt enough privacy while the swab was collected |  |  |  |  |  |  |  |
| 22. I am confident that I have collected the swab properly |  |  |  |  |  |  |  |

**Do you have any questions or comments? For example, did you miss or need certain things? If so, please write it down below.**

**After collection of the swab**

|  | Strongly disagree | Disagree | Slightly dis-agree | Sligthly agree | Agree | Strongly agree | I don’t know; n/a |
| --- | --- | --- | --- | --- | --- | --- | --- |
| 23. I was concerned that the cotton swab would contact unsanitary surfaces before it was placed into the tube |  |  |  |  |  |  |  |
| 24. I was concerned that I would spill liquid from the tube |  |  |  |  |  |  |  |
| 25. I found it difficult to break the cotton stick in the tube |  |  |  |  |  |  |  |
| 26. It was clear how the tube should be packed |  |  |  |  |  |  |  |
| 27. It was clear where the tube should be hand in |  |  |  |  |  |  |  |
| 28. I was concerned about the result of the test |  |  |  |  |  |  |  |

**Do you have any questions or comments? For example, did you miss or need certain things? If so, please write it down below.**

***Scenario 3:* sampling by a healthcare provider in the hospital**

**Collection of the cotton swab**

Immediately after your appointment with the urologist in the hospital, you were asked to collect a rectal swab. The following questions are about your experiences with this procedure.

Information about your experiences helps us -if necessary- to improve the information and logistics regarding the rectal swab collection.

Below we will ask you about your experiences **before** sampling, **during** sampling and **after** sampling.

**Please indicate the situation that applies to you:**

- The swab was collected by a doctor or nurse on the initiative of the doctor or nurse
- The swab was collected by a doctor or nurse at my request
- I collected the swab myself on the toilet in the hospital
- I collected the swab myself at home
- Otherwise, namely

**If needed, you can explain your answer here:**

**Your preferences:**

1. If you had the choice: would you rather collect the swab yourself or have it collected by a doctor or nurse?

- Self
- By doctor or nurse
- I have no preference

1. If you had the choice: would you rather collect the swab at home or in the hospital?
   - At home
   - In the hospital
   - I have no preference

**Before collection of the swab**

|  | Strongly disagree | Disagree | Slightly dis-agree | Sligthly agree | Agree | Strongly agree | I don’t know; n/a |
| --- | --- | --- | --- | --- | --- | --- | --- |
| 3. I found the explanation I received prior to collection of the swab clear |  |  |  |  |  |  |  |
| 4. I was afraid that I was physically incapable to collect the swab properly* |  |  |  |  |  |  |  |
| 5. I was confident that I could collect the swab properly* |  |  |  |  |  |  |  |
| 6. I was confident that the doctor/nurse was capable to collect the swab properly |  |  |  |  |  |  |  |
| 7. I was afraid that collecting the swab would hurt |  |  |  |  |  |  |  |
| 8. I was afraid that it would be difficult to collect the swab* |  |  |  |  |  |  |  |
| 9. I was afraid I couldn’t collect the swab on my own* |  |  |  |  |  |  |  |

** Question was only asked to patients who indicated that the swab was collected by the healthcare professional at the patient’s request.*

**Do you have any questions or comments? For example, did you miss or need certain things? If so, please write it down below.**

**During collection of the swab**

|  | Strongly disagree | Disagree | Slightly dis-agree | Sligthly agree | Agree | Strongly agree | I don’t know; n/a |
| --- | --- | --- | --- | --- | --- | --- | --- |
| 10. I was afraid that the cotton swab might break during the swab collection |  |  |  |  |  |  |  |
| 11. I experienced pain while the swab was collected |  |  |  |  |  |  |  |
| 12. I felt shame while the swab was collected |  |  |  |  |  |  |  |
| 13. I felt enough privacy while the swab was collected |  |  |  |  |  |  |  |
| 14. I am confident that the swab has been properly collected |  |  |  |  |  |  |  |

**Do you have any questions or comments? For example, did you miss or need certain things? If so, please write it down below.**

**After collection of the swab**

|  | Strongly disagree | Disagree | Slightly dis-agree | Sligthly agree | Agree | Strongly agree | I don’t know; n/a |
| --- | --- | --- | --- | --- | --- | --- | --- |
| 15. I was concerned about the result of the test |  |  |  |  |  |  |  |

**Do you have any questions or comments? For example, did you miss or need certain things? If so, please write it down below.**

**S5 - Patient questionnaires: free-text comments**

***Scenario 1:* self-sampling at home**

**Preferences:**

No remarks

**Before collection of the swab:**

*“I was not informed about what the investigation entailed. I received the instruction form in the hospital, had to read it in the waiting room and should have collected the swab immediately afterwards. I did not do this and went through the instruction at home (again) and handed in the swab a few days later. ”*

*“Perhaps it is a good idea to emphasize that the swab can be collected immediately in the hospital after it is handed out."*

*“I had no extensive instruction guide: swab collection – how to do.”*

**During collection of the swab:**

*“It is difficult to estimate how far you have inserted the swab. There should be a kind of ring around the stick that you feel when you are at the right depth.”*

*” You do something but you have no idea if you are doing it correct. Missed tip: it turned out to be useful to squat before insertion and not to stand (at least for me).”*

**After collection of the swab:**

*“There was a small amount of liquid inside the tube. It was unclear whether this was intended and whether it should remain in it. ”*

*“The use of the special transport system was problematic: it was unclear how to open and close the packaging.”*

*“There were many technical terms on the plastic bag of the transport system. These terms were not clear, e.g. pouch? blister? MMB?”*

*“The way in which the envelope should be closed was not entirely clear to me at first.”*

*“There are many similarities with the two yearly screening on colon cancer. It is unclear how to open the plastic bag (tear-off edge, adhesive strip). It was unclear how to open and close the packaging.”*

In one patient, the investigator (SCMT) observed that the swab arrived at the laboratory without the special transport system or laboratory form. A normal envelope provided with a Post-It had been used instead.

**General remarks:**

No remarks

***Scenario 2:* self-sampling in the hospital**

**Preferences:**

*“This should be done by a nurse.”*

*“Help from partner or nurse seems very pleasant to me.”*

*“Next time, I want that someone helps me.”*

*“I think the swab should be collected by a nurse.”*

*“Fine to do yourself.”*

*“No problem to do it yourself.”*

*“Given the questions, it would have been better if the nurse had clearly given me the choice whether I wanted to do it myself or let her do it for me. Then I would have let her do it: easily and certainly well.”*

*“I hope the swab collection went well, but next time I would prefer swab collection by a nurse.”*

**Before collection of the swab:**

Instructions:

*“Comes with a good description, it is easy to do.”*

*“Clear explanation. Often a matter of good reading.”*

*“It was not very clear to me when and where I had to collect the swab – so then I used a toilet in the hospital.”*

*“Better oral instruction would have been nice! Too little preparation time between the receipt and the collection of the swab, caught me by surprise!”*

*“The request for swab collection came quite unexpectedly. I would handle it better next time.”*

*“Explanation has been given by the nurse at the outpatient department Urology.”*

*“I did not read the instruction. The nurse clearly explained how to collect the swab.”*

*“Not difficult, read the description well in advance. Clear explanation with drawings ”*

*“Everything was explained properly.”*

*“The nurse was relaxed and gave good instructions: nothing to criticize. It was well explained and easy to do.”*

*“The urologist equated this action with the action of feeling the prostate. This is no comparison in terms of sensitivity or irritation. Collecting the swab should be explained as completely painless and simple, as if you were cleaning your ears with a cotton swab for example.”*

*“Explanation and guidance were very pleasant and clear.”*

**During collection of the swab:**

Location of swab collection:

*“It would be useful if there was a shelf in the toilet, in order to be able to impose the materials you have received.”*

*“The toilet in the hospital is too tight and had no opportunities to put down the other materials. That is a bit clumsy. I don’t know if I have been deep enough in the anus to get enough stool.”*

*“Toilets in the hospital don’t have the opportunity to put something down at a clean spot. The waste bin has a sloping top. This is bumbling if you don’t want to put the materials on the floor. The toilet floor was the only horizontal surface where I could put something down. I have experienced that as unsanitary.”*

*“Possibly a table or something to put the stuff on. Now it had to be put on the floor in the toilet. Not quite hygienic anyway.”*

*“I was referred to a toilet. Insufficient space to move next to the toilet. I was forced to deposit the tube and sticker on the lid of the toilet bowl. This is not convenient. Preferably in a separate room with sufficient comfort (by a nurse).”*

*“A) it took me 15 minutes to receive the explanation. B) a nurse reminded me at distance (with a full waiting room) that the toilet was not to the right but to the left at the end of the corridor. C) in the toilet was no possibility to put the materials down, so everything was on the floor! D) I was ordered to bring the swab after sampling to a room in the hospital: with my COPD gold C, almost D this took my very much effort. Very unpleasant treatment!!”*

*“On the toilet a shelf or something like that to put your stuff on. I found it not very hygienic on the toilet floor.”*

*“In the toilet there was no shelf were you could put your stuff on.”*

*“I found it uncomfortable and unsanitary to collect the swab on a public toilet. I missed a waste bin in the toilet to throw away the plastic bag and stick.”*

*“A disadvantage was that I had to collect the swab on one of the public toilets while I had the atrributes for the swab collection and an envelope with information. I was not sure where I could put the envelope with the information. There is no place on the toilet to put it except on the floor upright against the wall. It would be an idea to first collect the swab and then receive the information envelope.”*

*“Sampling was on the toilet in the hospital. Not exactly the most hygienic place!”*

*“I missed a toilet room where you could put things neatly. I had to put everything on the floor.”*

“*To collect the swab, you have to go to a toilet. These are not too spacious. That makes it uncomfortable to do this properly. One should be referred to a more spacious toilet.”*

“*There was no work surface in the toilet to put down the stuff. I was able to put everything on the toilet roll block, otherwise I had to put it on the floor.“*

*“Separate toilet.”*

“*There was no possibility to impose anything in the toilet. That had to be put on the ground.”*

“*Concerning sense of hygiene: you are supposed to do this in the toilet, where previously others were and you find the broken stick of the man before you. Have this done by the people in the room where I had the conversation with the nurse who explained me about this procedure.”*

*“The toilets are not well equipped to collect the swab. Different things have to be held or laid down at the same time, but only the towel machine could be used as a table. ”*

*“I was able to use the toilets in the central hall. I would prefer a toilet near the examination room in a quiet environment.”*

*“I found it was difficult to collect the swab on the toilet. The space was actually to small, to take off the jacket etc.”*

Help for swab collection:

*“My wife collected the swab in the disabled toilet. We were with two so it was easy to let my wife do it.”*

*“I thought little about the above because my wife collected the swab with ease.”*

*“My wife arranged it for me in the disabled toilet. Together in the disabled toilet is no problem.”*

*“I did not collect the swab myself. My partner did it, which was fine.”*

*“My wife did this for me in the disabled toilet in the hospital, because it is difficult for me to reach it myself.”*

Difficulties to insert the swab:

*“To get through the sphincter I had to moisten the anus with water.”*

*“The anus was on the dry side. As a result the cotton swab did not easily enter the anus. Possibly because I was obstipated.”*

*“It was difficult (probably I was a bit nervous) so there was blood on the rod.”*

*“Difficult to find the right place of insertion. Ditto how far.”*

*“It is quite difficult to insert because it is dry.”*

*“I think the swab collection was not optimal, because the insertion of the cotton swab didn’t work well due to the diameter (too thin).”*

Uncertainty about whether the swab was inserted deep enough:

*“The cotton swab had to be inserted 3 cm. Difficult to estimate this depth. If something would be attached to the cotton swab that wouldn’t allow you to go deeper, it would have been easier.”*

*“To insert the cotton swab ‘a few centimeters’ is difficult to measure. Maybe make a tactile point on the stick?”*

*“I don’t know if I collected the swab deep enough.”*

*“I collected the swab myself in the hospital after advice from a nurse. However, she indicated that the anus had to be sampled, while I told her that I thought that this should be 2 cm in the intestine. I (unfortunately) followed the advice of the nurse. When I read the instruction at home it clearly stated that the swab should have been collected 2-3 cm in the intestine.”*

*“I do not know if I went deep enough.”*

*“I found It difficult to determine if the depth was good. I stopped when I experienced some pain. I think I had the right depth.”*

*“I did not know how far to insert the swab and when turning it was sensitive.”*

Uncertainty about whether the swab was collected properly:

*“There was almost nothing on the swab.”*

*“I did not see anything on the tip, even after inserting the cotton swab several times. So I was unsure whether there was enough research material on the cotton swab. Nevertheless, after several attempts, I put the cotton swab in the tube, closed it and delivered it at the laboratory.”*

*“Poo was not visible, therefore I inserted the cotton swab a second time.”*

*“The swab was collected after an internal examination/touché whereby vaseline was used. This also may have contaminated the swab.”*

*"I collected the swab immediately after an internal examination, so there was also some kind of lubricant in the anus."*

*“I wonder if the swab collection was successful.”*

*“I did it myself, very uncomfortably. Good? I don’t know.”*

*“There was blood in the stool, which made me doubt whether it went well.”*

*“Is it useful/necessary to repeat the sampling? My biopsy is scheduled for Tuesday 11 February.”*

*“I found it difficult because I did not know if it went well.”*

*“I don’t know if I did it right, I think so.”*

*“I prefer to do it myself, but doubt whether it was done well. For example: was there enough stool on the swab? Have I turned it enough?”*

**After collection of the swab:**

Problems with the packaging:

*“Opening the plastic bag is not properly indicated and takes a lot of strength. It seemed that the back of the sticker was not present. Therefore the back of the sticker was difficult to remove.”*

*“I had problems with opening the plastic package.”*

“*I found it very difficult to open the plastic without scissors or knife and there is no place to put the tube to have one hand free.”*

*“The bag was difficult to open.”*

*“It was unclear whether the barcode sticker should be placed on the tube.”*

*“I put the sticker that was included on the tube: my wife doubted whether this was correct, I was convinced that it should be done like this.”*

Location of returning the swab after sampling:

*“I had to walk to the public toilets and with the stuff through the hospital to deliver it at another place. I would have preferred to do the smear near the place the swab had to be delivered.”*

*“The receiving laboratory was not informed”.*

*“Because of the hassle in the toilet, I only discovered the plastic bag with the address when I was already in the car. When I delivered the tube in the hospital, no one was aware of the study.”*

*“At the return desk people did not know immediately where the sample had to go to.”*

*“The return desk in the hospital was not occupied for 10 minutes, so I started to doubt whether I was at the right place. A simple sign or note that indicated, ‘I will be right back’ would have removed that doubt.”*

*“It was a bit of a search for the lab. Handing the sample in where I received it would have been easier.”*

*"The location where I had to return the cotton swab was a bit confusing. I did not exactly know where to go.”*

**General remarks:**

*“It was no problem for me.”*

*“Collecting the swab was nothing. It was very easy.”*

*“I have no problem with anything.”*

*“The tip of the cotton swab should be rounded.”*

*“I did not go to the toilet in the morning due to hard stools, a lot of sitting lately. The blood on the cotton swab is possibly due from internal hemorrhoids.”*

***Scenario 3:* sampling by a healthcare provider in the hospital**

**Preferences:**

*"I wouldn't have bothered with it at all to collect the swab myself, but was very kindly helped by the nurse who informed me."*

*“The swab was collected immediately by the person who asked me to participate in the study. I was also allowed to do it at home, but it was much easier that she did it and that the swab could be sent directly to the laboratory. Otherwise, I would have to come back to return the swab to the hospital.”*

**Before collection of the swab:**

*"In my opinion, everything is explained sufficiently and clearly!"*

*“I thought that no swab had been collected. I only found out after consulting Mrs. Tops of the Radboud university medical center.”*

**During collection of the swab:**

*“During the consultation with the doctor, a nurse (?) collected the swab so lightly that I wonder if it was enough for a good examination. No fear of… and no pain when sampling, but I also received no explanation.”*

*“I couldn't insert the cotton swab myself, and then asked if they would help me. It was very difficult for me with the cotton swab, it took me at least 10 minutes, but I did not manage to do it, therefore I asked for help.”*

*“I collected a swab twice, the first time was very painful, the second painless.”*

**After collection of the swab:**

No remarks

**General remarks:**

No remarks

**S6: Instruction form: rectal swab collection prior to prostate biopsy**

During a prostate biopsy, a healthcare provider removes small samples of tissue from the prostate using a thin needle. Because the prostate is punctered through the rectum, there is a small risk of prostate or urinary tract infections after prostate biopsy, caused by intestinal bacteria. To prevent infections after prostate biopsy, a short course of antibiotics is started prior to prostate biopsy. Unfortunately, some people have intestinal bacteria that are insensitive (resistant) to the standard antibiotics used prior to prostate biopsy. Therefore, we want to ask you to collect a rectal swab. In this way, the sensitivity of the intestinal bacteria to certain types of antibiotics can be examined in your stool which helps us to determine which type of antibiotics are most suitable for you to use prior to prostate biopsy.

Option 1 (self-sampling at home): We would ask you to collect the rectal swab at home no later than [date].

Option 2 (self-sampling in the hospital): We would ask you to collect the rectal swab in the hospital immediately after the outpatient visit to your urologist

Below you can find instructions on how to collect this swab. Please read these instructions carefully first.


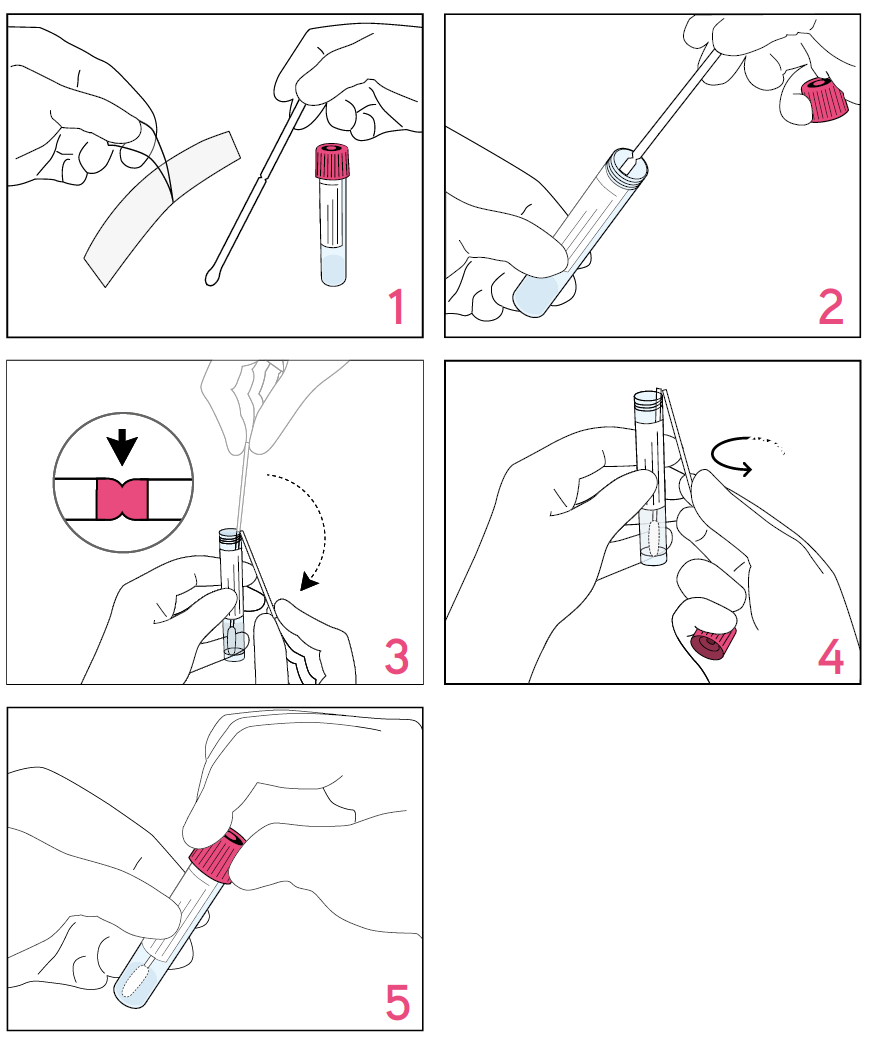

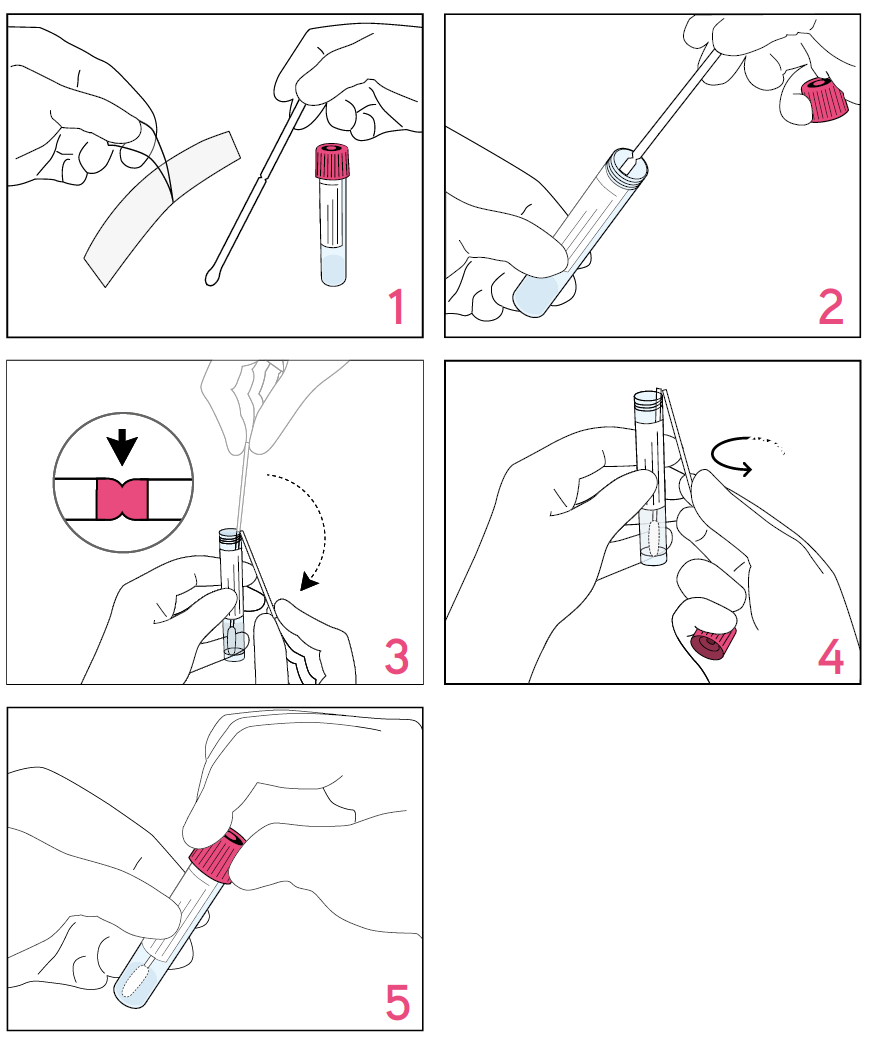


1. Open the package and remove the swab and tube from the package. **Avoid touching the swab below the pink breaking point.** Insert the swab into the intestine through the anal sphincter. Rotate the swab 3x **in** the intestine. The smear has been successfully performed if faecal material is visible on the tip of the swab. If stool is not visible, reinsert until the swab is coated.

N.B. the presence of lubricant or vaseline in the rectum (for example after internal examination) does not affect the smear.

N.B. if desired, use vaseline to ensure easy insertion of the swab.

2. Remove the screw cap from the tube.

Liquid is present in the tube. Be careful not to spill it.

Insert the swab all the way to the bottom of the tube.


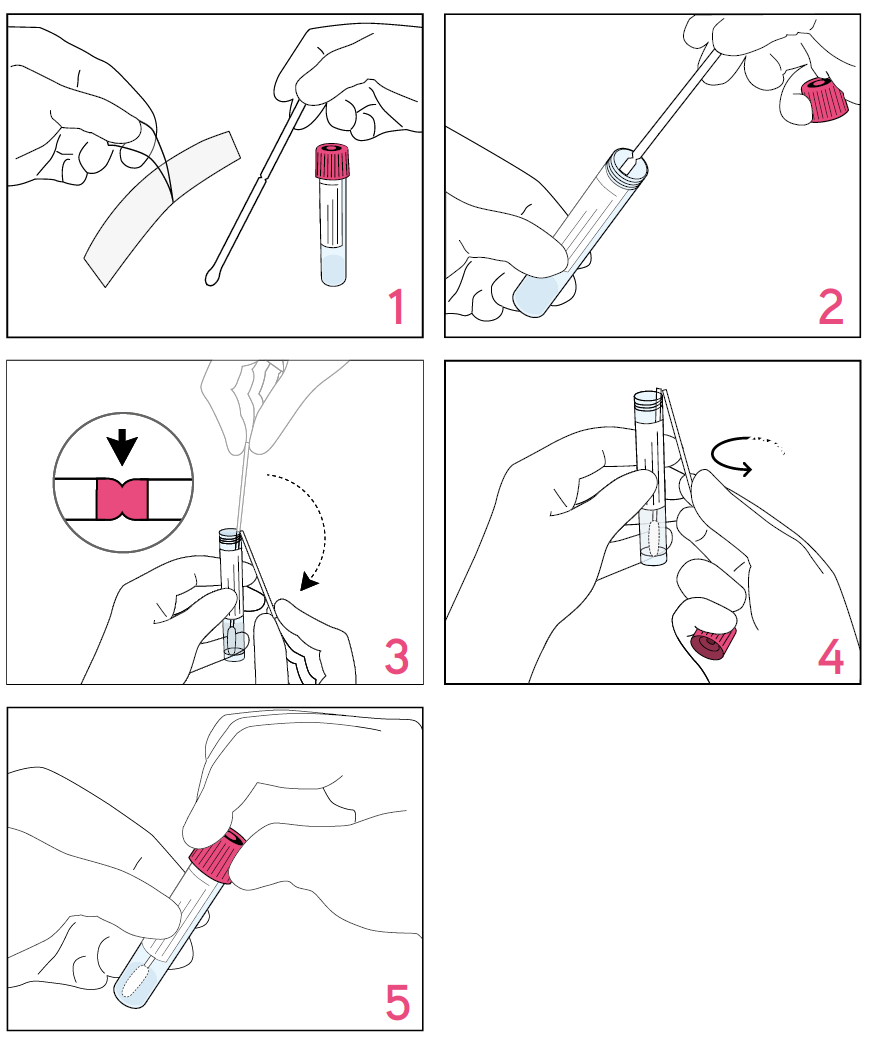


3. Hold the tube away from your face. Break the swab in the tube. There is a pink breaking point on the swab.

4. If you have broken the swab, you can dispose the end of the swab. The part of the swab with the cotton ball must be left inside the tube.

5. Screw the cap on tightly to prevent leakage.

**Apply the attached patient identification label on the tube.**

Option 1 (self-sampling at home): Insert the tube in the absorbent pouch. Thereafter, insert the absorbent pouch (with the tube) into the plastic seal bag and remove the strip from the flap closure to seal the bag. Then, place the plastic seal bag in the protective cardboard. Finally, insert the protective cardboard into the blue plastic envelope. Make sure the laboratory form is also present in the blue plastic envelope. Seal the envelope by removing the strip from the flap closure. No stamp is required.

Option 2 (self-sampling in the hospital): Insert the tube in the plastic bag. Make sure the laboratory form is also present in the plastic bag. Seal the plastic bag.


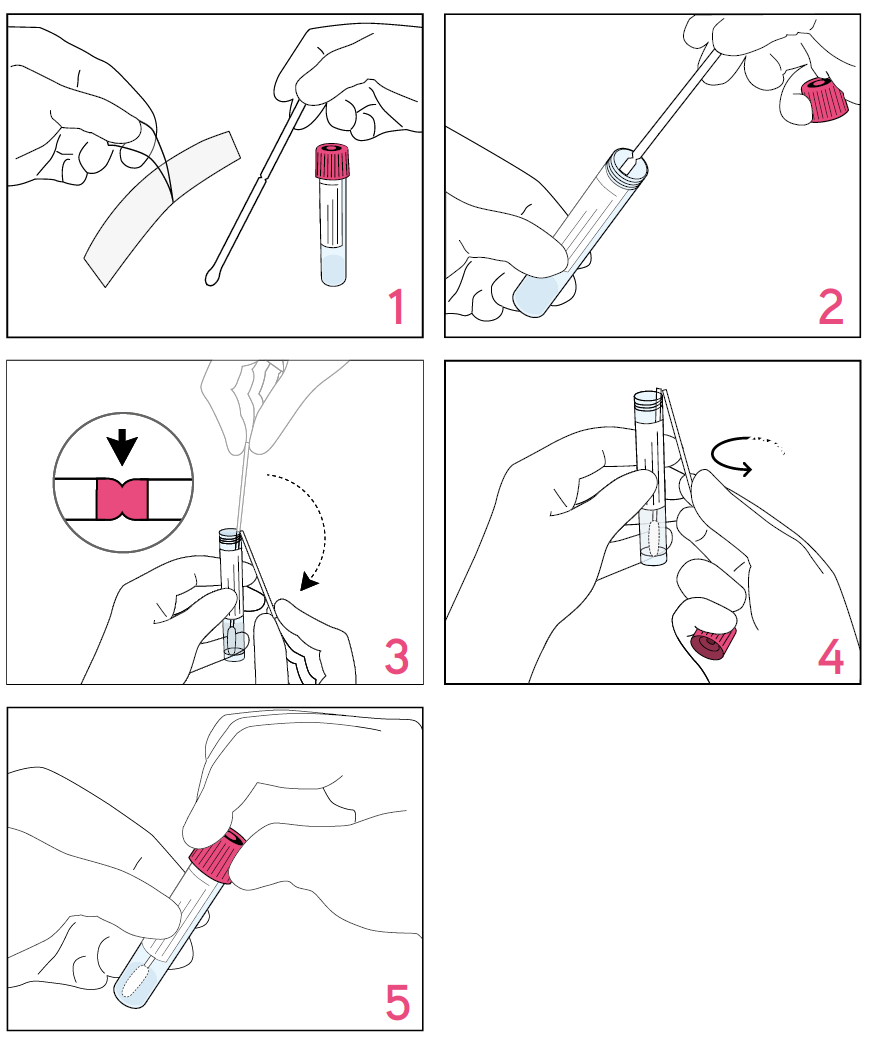

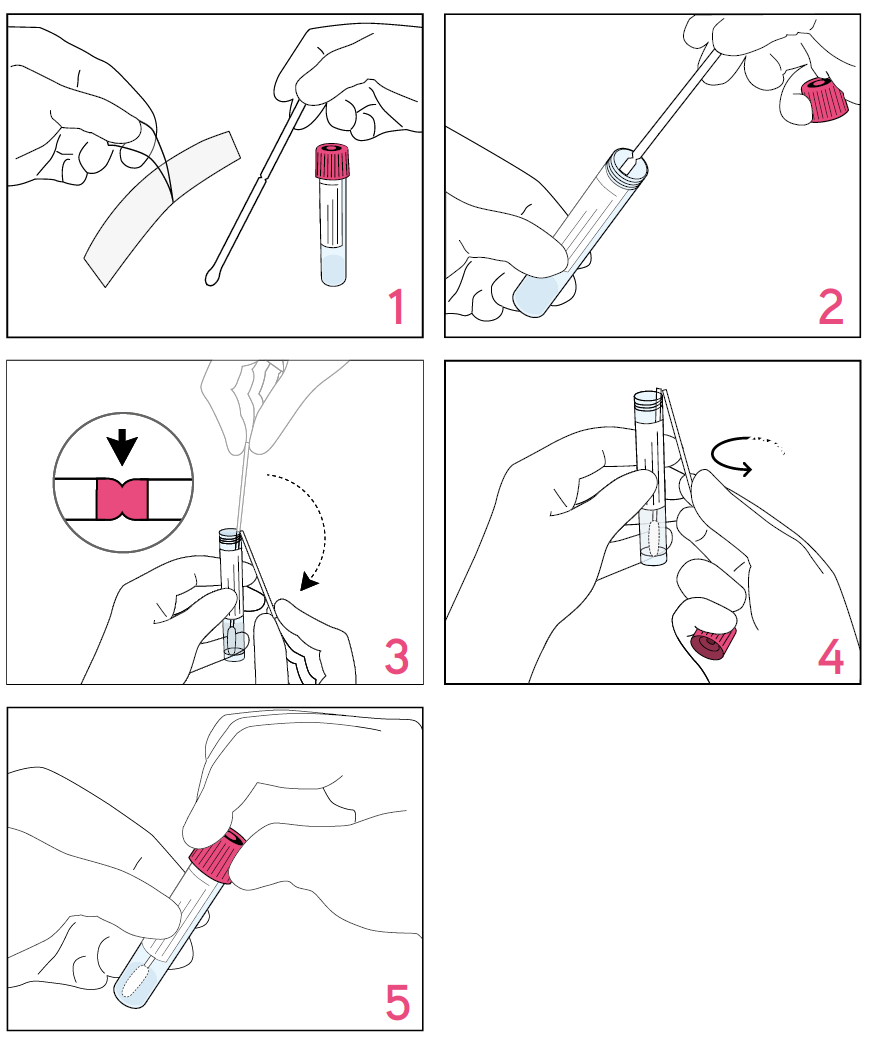


Option 1 (self-sampling at home): Return the special transport system with the tube and the laboratory form as soon as possible after sampling (no later than 24 hours after sampling) by post. Keep the tube in the refrigerator before sending it per post. Due to the postal delivery, you can only post it from Monday to Thursday evening 5:00 PM.

Option 2 (self-sampling in the hospital): Deliver the tube and the laboratory form in the sealed plastic bag directly to [location] of the [name] hospital, [route]. Opening hours: [….]

**If you have any questions or concerns please contact [contact details].**
